# Anti-lung cancer effect of paclitaxel solid lipid nanoparticles delivery system with curcumin as co-loading partner in vitro and in vivo

**DOI:** 10.1080/10717544.2022.2086938

**Published:** 2022-06-24

**Authors:** Chao Pi, Wenmei Zhao, Mingtang Zeng, Jiyuan Yuan, Hongping Shen, Ke Li, Zhilian Su, Zerong Liu, Jie Wen, Xinjie Song, Robert J. Lee, Yumeng Wei, Ling Zhao

**Affiliations:** a Key Laboratory of Medical Electrophysiology, Ministry of Education, School of Pharmacy of Southwest Medical University, Luzhou, Sichuan, P. R China; b Luzhou Key Laboratory of Traditional Chinese Medicine for Chronic Diseases Jointly Built by Sichuan and Chongqing, The Affiliated Traditional Chinese Medicine Hospital of Southwest Medical University, Luzhou, Sichuan, P. R. China; c Central Nervous System Drug Key Laboratory of Sichuan Province, Southwest Medical University, Luzhou, Sichuan, P. R. China; d Clinical Trial Center, The Affiliated Traditional Chinese Medicine Hospital of Southwest Medical University, Luzhou, Sichuan, P. R China; e Central Nervous System Drug Key Laboratory of Sichuan Province, Sichuan Credit Pharmaceutical CO., Ltd, Luzhou, Sichuan, P. R. China; f Key Laboratory of Biorheological Science and Technology, Ministry of Education, College of Bioengineering, Chongqing University, Chongqing, Shapingba, P. R. China; g School of Biological and Chemical Engineering, Zhejiang University of Science and Technology, Hangzhou, Zhejiang, P. R. China; h Department of Food Science and Technology, Yeungnam University, Gyeongsan-si, Republic of Korea; i Division of Pharmaceutics and Pharmacology, College of Pharmacy, The Ohio State University, Columbus, Ohio, USA

**Keywords:** Paclitaxel, curcumin, solid lipid nanoparticles, combination therapy

## Abstract

The main aim of this study was to improve the therapeutic potential of a paclitaxel (PTX) and curcumin (CU) combination regimen using solid lipid nanoparticles (SLNs). PTX and CU were successfully co-encapsulated at a predetermined ratio in SLNs (PC-SLNs) with high encapsulation efficiency (CU: 97.6%, PTX: 95.8%), appropriate particle size (121.8 ± 1.69 nm), small PDI (0.267 ± 0.023), and negative zeta potential (–30.4 ± 1.25 mV). Compared with PTX or the combination of CU and PTX (CU + PTX), PC-SLNs can greatly reduce the dose of PTX while still achieving the same therapeutic effect on four cancer cell lines, among which the inhibitory effect on A549 lung cancer cells was the strongest. PC-SLNs improved the area under the curve (CU: 1.40-fold; PTX: 2.88-fold), prolonged the residence time (CU: 6.94-fold; PTX: 2.51-fold), and increased the half-life (CU: 5.62-fold; PTX: 6.46-fold), achieving long circulation. PC-SLNs were used to treat lung cancer in a nude mouse xenograft tumor model and the tumor suppression rate reached 78.42%, while those of PTX and (CU + PTX) were 40.53% and 51.56%, respectively. As PC-SLNs can prevent P-glycoprotein efflux, reverse MDR and downregulate the NF-κB pathway. PC-SLNs are a potential antineoplastic agent that is more effective and less toxic in treating lung cancer.

## 1. Introduction

Cancer is among the most destructive diseases, according to statistics, nearly 20 million new cancer cases and nearly 10 million cancer deaths occur worldwide. The deaths caused by lung cancer total more than 1.8 million cases, accounting for 18% of the total cancer deaths; thus, it ranks first in terms of cancer deaths (Ferlay et al., 2021; Sung et al., 2021). Despite the varied types of therapeutic modalities available, chemotherapy based on anticancer small molecule cytotoxic drugs is the most common approach. Paclitaxel (PTX), which is isolated from the bark of Taxus brevifolia, is one of the most widely used chemotherapeutic agents to treat lung cancer. Currently, commercially available PTX preparations include PTX injection and PTX albumin nanoparticles (Chen et al., 2021). Polyoxyethylene castor oil and absolute ethanol were used to prepare PTX injections that can cause allergic reactions. Although the incidence of allergic reactions of PTX albumin nanoparticles is very low, a higher rate of myelotoxicity is found in clinical trials, limiting its application (Adrianzen Herrera et al., 2019; Jiménez-López et al., 2019).

Recent studies have found that PTX in combination with natural drug monomers, such as curcumin (CU), triptolide, and honokiol, can reduce the dose of PTX needed and increase the therapeutic effect (Liu et al., 2018; Guo et al., 2020; Li et al., 2021). Furthermore, numerous preclinical studies have shown that a combination of PTX and CU may be an effective strategy to synergistically improve the therapeutic efficacy of cancer therapeutics and reverse multidrug resistance (MDR) (Calaf et al., 2018; Ashrafizadeh et al., 2020^;^ Lee et al., 2020). On the one hand, studies have shown that a PTX-CU combination can not only downregulate Bcl-2 (an anti-apoptotic protein) but also upregulate Caspase-3 and Bax (two pro-apoptotic proteins) to promote apoptosis in a synergistic manner, as well as the cancer metastasis markers, including HIF-1α, VEGF, and MMP 2, can be downregulated through treatment of PTX-CU combination to inhibit proliferation and metastasis (Berrak et al., 2016; Quispe-Soto & Calaf, 2016; Yuan et al., 2017; Calaf et al., 2018; Mu et al., 2020; Yan et al., 2020; Zhang et al., 2020). On the other hand, CU can downregulate the MDR-1 and ALDH-1 gene expression to decrease PTX drug resistance and can decrease the expression of NF-κB and P-gp to strengthen the sensitivity of breast cancer cells to PTX to reverse MDR (Bava et al., 2011; Ruiz de Porras et al., 2016; Attia et al., 2020). The synergistic effects of the PTX and CU combination *in vitro* have been demonstrated by these mechanisms illustrate.

Unlike *in vitro* assays, in which drug exposure to the tumor cells can be easily controlled and quantified, the same control is difficult to achieve *in vivo*. Similarly, many drugs in development show good results in *in vitro* experiments but do not exhibit the same effect *in vivo*. At present, a crucial issue that needs to be addressed in drug combination therapy is how to transfer the synergistic effects observed *in vitro* to benefits that can be observed *in vivo* (Mayer & Janoff, 2007; Tardi et al., 2009). The use of a ‘cocktail’ regimen is a common strategy; namely, two or more drugs are administered after physical mixing (Baghbani & Moztarzadeh, 2017; Zhang et al., 2017; Zhu et al., 2019). Although this regimen is convenient, it has certain limitations. For example, different drugs have different pharmacokinetics, tissue distributions, and cell membrane permeabilities, leading to inconsistent drug intake at the tumor site, and identifying the best drug synergistic ratio *in vivo* from *in vitro* experiments is difficult, thereby leading to the failure of combination treatment strategies.

To further improve the pharmaceutical profile of the PTX and CU combinations, an appropriate co-loading delivery system needs to be selected (Utreja et al., 2011; Zhou et al., 2019). Many nano delivery systems have been developed for the delivery of anticancer drugs to prolong their circulation *in vivo*, enhance their accumulation in tumor tissue, and increase tumor cell-specific uptake (Cui et al., 2013; Joseph et al., 2020^;^ Xu et al., 2021). Solid lipid nanoparticles (SLNs) can boost the stability of drugs and prolong their half-life and average residence time *in vivo*, which can facilitate the sustained and controlled release of drugs and improve treatment efficacy (Madan et al., 2013; Tao et al., 2019; Valdes et al., 2019; Koroleva et al., 2022). In addition, SLN can release drugs via a target aggregation in tumor tissue, and the unique microenvironment of the tumor can influence the lipid membrane of the SLN (Matsumura & Maeda, 1986; Nielsen et al., 1994; Maeda et al., 2009). In this way, SLNs coloaded with PTX and CU could be used to efficiently enrich drugs in the tumor tissue, reduce the drug dose, and prevent damage to normal organs or tissues, and they have the potential to reverse MDR in tumors (Tang et al., 2017; Zhao et al., 2017; Zhou et al., 2018; Qiao et al., 2019).

In the previous studies of drug combination index screening, we selected a ratio (PTX: CU = 1: 2) that has a beneficial synergistic effect with the combinational index predictive values of IC_75_ were below 0.5 on cancer cells and it has an excellent safety profile in normal hepatocytes (L02) (Li, 2019; Feng et al., 2020). The goal of this study was to investigate whether the synergistic *in vitro* effect of PTX and CU can be transferred to *in vivo* effects and whether it can improve anticancer drug combination treatments based on SLN. Therefore, we prepared SLNs containing PTX and CU at a ratio of 1:2 (PC-SLN) and screened out the most sensitive cell line (A549). To further clarify the molecular mechanism of the combination therapy, the expression of NF-κB in A549 cells was evaluated by western blotting analysis. Moreover, synergistic effects were also verified by *in vivo* experiments.

## 2. Materials and methods

### 2.1. Materials

Curcumin (CU, purity ≥ 98%) and paclitaxel (PTX, purity ≥ 98%) were purchased from Chengdu Best Reagent Co., Ltd. Hydrogenated soybean phospholipids (HSPC) and 1,2-distearoyl-sn-glycero-3-phosphoethanolamine-N-[methoxy (polyethylene glycol)-2000] (DSPE-PEG2000) were purchased from Lipoid GmbH. Polyvinyl pyrrolidone k15 (PVPk15) was purchased from Shandong Yousuo Chemical Technology Co. Ltd.

### 2.2. Cell culture

RPMI 1640 or DMEM medium, fetal serum, 0.25% trypsin, and 100 units/mL penicillin-streptomycin were purchased from Thermo Fisher Company. The 3-(4,5-dimethyl-2-thiazolyl)-2,5 diphenyl-2-H-tetrazolium bromide (MTT) reagent was purchased from Luzhou Renkang Biotechnology Co., Ltd. The NF-κBp65, Bcl-2, Bax, P-gp, and β-actin antibodies were purchased from Santa Cruz Company.

SMMC-7721, MCF-7, CaCO2 and A549 cells were obtained from the Shanghai Cell Bank of China, L02 cells were obtained from the Kunming Cell Bank of China, and the cells were cultured in RPMI 1640 or DMEM medium, which was supplemented with 100 units/mL penicillin-streptomycin and 10% fetal calf serum, at 37 °C in a 5% CO_2_ incubator (HEPA class 100, Thermo, GER). Cells in the exponential growth phase were used for all of the experiments.

### 2.3. Animals

Nude BALB/c mice that were four to six weeks old, half female and half male, and 18 ± 2 g were obtained from Spitford Biotechnology Company with the following Laboratory Animal License: SCXK (Jing) 2016-0002. Animal feeding occurred at a temperature of 20 ± 2 °C and relative humidity of 40–60% in the IVC Animal Feeding Room at the Laboratory Animal Center of Southwest Medical University

Albino New Zealand rabbits (1.5 ± 0.1 kg) were supplied by the Laboratory Animal Center of Southwest Medical University (approval number: SYXK (Chuan) 2018-065). The rabbits were housed in a pathogen-free environment with free access to food and water. The studies involving animals were approved by the Ethics Committee of Southwest Medical University (No 2015DW040).

### 2.4. Preparation, optimization, and characterization of PC-SLN

Solid lipid nanoparticles loaded with PTX and CU (PC-SLN) were prepared by using the film dispersion method (Tai et al., 2020). Ultrasound was used to dissolve CU (5 mg), PTX (2.5 mg), HSPC (70 mg), PVP_K15_ (8 mg), and DSPE-PEG2000 (15 mg) in 3 mL CHCL_3_. Then, the solution was transferred to a round bottom flask (50 mL) and was spun and evaporated at 45 °C to produce a film. Then, 6 mL deionized water containing 1% sucrose (w/v) was added to the round bottom flask and probe ultrasonication was performed for 5 min. The final solution was lyophilized by freeze-dryer (LGJ-18C; Fourth-Ring Science Instrument plant Beijing Co., Ltd., Beijing, CHN) at −50 °C 0.9 Pa for 24 h to obtain PC-SLNs.

Based on the results of a single-factor experiment, an orthogonal experimental design was used to optimize the formulation of PC-SLN. Four factors of PC-SLN including the amount of CU + PTX (A), HSPC (B), DSPE-PEG2000 (C), and ultrasonic intensity (D), and each factor had three levels arranged according to an L_9_(3^4^) orthogonal experiment table (Table 1). The encapsulation (EE%) of CU (S1), EE% of PTX (S2), drug loading (DL%) of CU (S3), DL% of PTX (S4), and PDI(S5) were taken as evaluation indexes. The weighted analysis method was used for the comprehensive analysis of the indicators. S1, S2, S3, S4 and S5 were integrated with the coefficients of 40%, 40%, 5%, 5% and 10%, respectively. S indicates the comprehensive scoring index, calculated as follows:

$$S=\frac{S1}{{S1}_{\max}}\times 40\%+\frac{S2}{{S2}_{\max}}\times 40\%+\frac{S3}{{S3}_{\max}}\times 5\%+\frac{S4}{{S4}_{\max}}\times 5\%+\frac{S5}{{S5}_{\max}}\times 10\%$$

**Table 1.** L_9_ (3^4^) Orthogonal design table (*n* = 3).

No.	A (mg)	B (mg)	C (mg)	D (%)	S1	S2	S3	S4	S5	S
1	6 + 3	70	20	25	89.93	88.34	3.09	1.43	0.341	74.64
2	6 + 3	65	25	30	89.60	87.71	3.15	1.51	0.305	75.72
3	6 + 3	60	30	35	90.24	89.78	3.27	1.52	0.275	77.89
4	5 + 2.5	70	25	35	94.83	93.26	2.86	1.41	0.247	82.61
5	5 + 2.5	65	30	25	92.76	92.72	2.71	1.37	0.230	80.15
6	5 + 2.5	60	20	30	92.71	92.97	2.73	1.36	0.207	80.93
7	2.5 + 1.25	70	30	30	95.14	94.38	1.37	0.65	0.228	77.51
8	2.5 + 1.25	65	20	35	94.56	94.46	1.38	0.68	0.255	76.63
9	2.5 + 1.25	60	25	25	90.56	91.92	1.35	0.69	0.252	73.93
K1	228.26	234.77	232.20	228.72						
K2	243.70	232.50	232.26	234.16						
K3	228.06	232.75	235.56	237.13						
R	5.15	0.76	1.12	2.80						

The intuitive and variance analysis were used to determine the best formulation.

The zeta-potential particle size and polydispersity index (PDI) of PC-SLNs were characterized by a Malvern Zetasizer Nano instrument (ZS, Malvern, Worcestershire, UK). The EE% and DL% were determined as follows. CU and PTX are both hydrophobic molecules, thus, with low-speed centrifugation, PC-SLNs with smaller particle sizes cannot form a precipitate, but the drug, which exists in a free state, precipitates in a crystal form. Therefore, to evaluate the EE% and DL%, low-speed centrifugation (8000 rpm, 8 min) was selected to separate free PTX or free CU. The same volume of PC-SLN suspension before and after low-speed centrifugation was measured, demulsified, and diluted with methanol. The lyophilized PC-SLN powder was accurately weighed, suspended in methanol. The same volume of PC-SLN suspension before and after low-speed centrifugation was measured and demulsified by ultrasound (200 W, 40 kHz, 4 min). After the sample was filtered and diluted with methanol, Then, a filtered sample (20 μL) was injected into a reversed-phase C18 HPLC column (4.6 × 250 mm, Phenomenex, America) with a particle size of 5 μm. The CU and PTX contents were determined by high-performance liquid chromatography (HPLC) (1260 Infinity II, Agilent Technologies, USA). The separation was performed at 30 °C. The flow rate of the mobile phase was 1.0 mL/min, 0–10.2 min [acetonitrile-0.1% phosphoric acid solution (55:45, v/v)], and the samples were detected at 425 nm; then, the mobile phase was changed to [acetonitrile-0.1% phosphoric acid solution (40:80, v/v)] at 10.2–14.2 min, and the samples were detected at 227 nm; and finally, the mobile phase was changed to [acetonitrile-0.1% phosphoric acid solution (55:45, v/v)] at 14.2–19 min, and the samples were detected at 425 nm. The EE% and DL% were calculated as follows:

$$\mathrm{Encapsulation}(\text{EE\%})=\frac{W_{e}}{W_{t}}\times 100\%$$

$$\text{Drug loading }(\text{DL\%})=\frac{W_{t}}{W_{0}}\times 100\%$$

where W_e_ was the amount of drug loaded in CUD-SLN, W_t_ is the total amount of drug in CUD-SLN, and W_0_ is the total weight of SLN.

### 2.5. In vitro cytotoxicity assay

To examine the inhibitory effect of the CU and PTX preparations on cancers, SMMC-7721, MCF-7, CaCO2, A549, and L02 cells were inoculated into 96-well plates at a density of 5 × 10^3^ cells/well/100 μL. After being cultured for 24 h, CU solutions (3.125, 6.25, 12.5, 25, 50, and 100 μg/mL), PTX solutions (1.563, 3.125, 6.25, 12.5, 25, and 50 μg/mL) and groups with combinations of CU and PTX (2:1, m/m) and PC-SLN were added to 96-well plates at gradient concentrations and incubated for 72 h. Then, 20 μL of MTT (5 mg/mL) was added to each well, and the cells were incubated for another 4 h at 37 °C in the dark. The aliquots were removed, and the remaining crystals (formazan precipitates) were solubilized with 150 μL of DMSO. The absorbance (A) was measured at 490 nm. The cell growth inhibitory rate was calculated as follows:

$$\text{Cell growth inhibition rate }(\%)=\frac{1-(A_{\mathrm{experimental}}-A_{\mathrm{blank}})}{A_{\mathrm{control}}-A_{\mathrm{blank}}}\times 100\%$$

SPSS 17.0 software (IBM SPSS Statistics, Chicago, IL, USA) was used to calculate the half-maximal inhibitory concentration (IC_50_) values of the drugs.

### 2.6. Cell apoptosis assay and cell cycle

A549 cells were inoculated into 6-well plates at a density of 1 × 10^5^ cells/well/mL. The cells were cultured for 24 h, and CU, PTX, (CU + PTX), and PC-SLN solution (1, 5, and 15 µg/mL) were added to the 6-well plates. The control group without drugs was set up at the same time. After culturing for 48 h, the cells were collected and washed once with PBS. Then, 500 μL binding buffer, 5 μL Annexin V/FITC, and 5 μL PI were added to every sample in a 5 mL culture tube and mixed well at room temperature in the dark for 10 min. Flow cytometry was used to obtain measurements within 1 h, and the excitation and emission wavelengths were 488 and 530 nm, respectively. The FITC channel (FL1) detected green fluorescence, and the PI channel (FL2) detected red fluorescence. In the control groups, only 5 μL Annexin V/FITC or PI was added. Finally, flow cytometry (FACSCalibur, BD Biosciences, San Jose, CA, USA) and FlowJo software (BD Biosciences, San Jose, CA, USA) were used to determine the apoptosis rate.

A549 cells were inoculated into six-well plates at a concentration of 4 × 10^5^ cells/well. The cells were incubated with 50 mg/mL CU, PTX, (CU + PTX), and PC-SLN for 24 h. All cells were digested with trypsin, washed twice with PBS, and then fixed in PBS with 70% dry ice-ethanol for 30 min at 20 °C. Finally, the cells were washed twice with PBS, treated with RNase A (10 mg/mL), and then suspended in 50 mg/mL PI for dyeing. Flow cytometry was used to determine the cell cycle distribution (Zhang et al., 2019).

### 2.7. Protein expression

The test was divided into the following groups (with the same drug concentration): group one, blank control (RPMI 1640 medium containing the same concentration of DMSO); group two, CU alone; group three, PTX alone; group four, (CU + PTX); and group five, PC-SLN. Then, A549 cells (up to a density of 90%) were seeded at 1 × 10^6^ cells into 10 cm^2^ Petri dishes, incubated for 24 h and treated with drugs in the above five groups for 48 h. Then, the culture media were discarded, and the cells were washed with cold PBS buffer twice to be harvested. Cell pellets were disrupted with cell RIPA buffer and collected after centrifugation at 16,000 rpm for 10 min at 4 °C, and the lysates were centrifuged at 15,000 rpm for 10 min at 4 °C to collect the cytoplasmic and nuclear proteins. The protein concentrations were determined by the phenyl methane sulfonyl fluoride (PMSF) method. Protein samples (30 μL each) were loaded on SDS–PAGE (sodium dodecyl sulfate-polyacrylamide gels), separated by electrophoresis and subsequently transferred onto NC membranes. Nonspecific binding was blocked with 5% skimmed milk in TBST (5 mmol/L Tris-HCl, 136 mmol/L NaCl, and 0.05% Tween-20, pH 7.6) for 1 h. The membranes were cultured with primary antibodies against Bax (affinity, AF0120, 1:1000), NF-Κbp65 (affinity, AF5006, 1:1000), Bcl-2 (affinity, AF6139, 1:1000), P-gp (abcam, ab170904, 1:1000) or β-actin (abclonal, AC026, 1:100000) overnight at 4 °C. Then, the membranes were washed three times with 1× TBST, incubated with secondary antibodies (abcam, ab6721, 1:1000 dilution) at room temperature for 2 h and washed three times with 1× TBST. Protein bands were visualized by an enhanced chemiluminescence (ECL) system (Amersham Biosciences, Piscataway, NJ, USA). The gray values of the Bax, NF-κBp65, Bcl-2, P-gp, and β-actin proteins were measured by Quantity One software (Bio-Rad, Hercules, CA, USA) (Behbahani et al., 2017).

### 2.8. Pharmacokinetic studies

The rabbits for the pharmacokinetic study underwent fasting and had free access to water for over 24 h before the experiment. The rabbits (1.5 ± 0.1 kg) were randomly divided into two groups, each containing five rabbits. CU (16 mg/kg) and PTX (8 mg/kg) powder (2:1, m/m) were used, and PC-SLN (16 mg/kg of CU, 8 mg/kg of PTX) was used. All agents were administered by intravenous marginal ear vein injection. Approximately 1 mL of blood was collected from the marginal ear vein using a heparin-coated syringe at 5, 15, 30, and 45 min and 1, 2, 4, 6, 8, 12, and 24 h after injection. The blood samples were centrifuged at 5000 rpm for 10 min to obtain supernatant plasma. 475 μL of plasma was added to 25 µL of acetate buffer (pH 3.6). After vortexing for 30 s, 3 mL of extract solvent [methanol-ethyl acetate 10:90, v/v] was added to the mixture, and the mixture was vortexed again for 3 min and then centrifuged at 8000 rpm for 3 min. Then, 2.4 mL of supernatant was removed, and the organic solvent was evaporated by a nitrogen flow. The dried residue was dissolved in a 100 μL mobile phase, followed by centrifugation at 8000 rpm for 10 min. The concentration of CU or PTX in the plasma sample was measured using the HPLC analytical method described previously. Pharmacokinetic data were determined using a noncompartmental method based on the statistical moment theory (Zhang et al., 2019).

### 2.9. Efficacy evaluations

Approximately 2.5 million A549 tumor cells were inoculated subcutaneously into the right forelimb of 4-week-old nude mice. The tumor volume (V) was determined according to the equation V = 0.5 ×a × b^2^, where a was the maximum perpendicular diameter and b was the minimum perpendicular diameter, using dial caliper measurements. When the tumor volume reached 100 ± 20 mm^3^, tumor-bearing nude mouse models were selected for subsequent experiments.

Twenty-five tumor-bearing nude mice were randomly divided into five groups with 5 mice in each group as follows: negative control group (saline); CU group, CU 16 mg/kg; PTX group, PTX 8 mg/kg; (CU + PTX) group, CU 16 mg/kg and PTX 8 mg/kg; and PC-SLN group (calculated by CU), 16 mg/kg. All groups were injected with 0.05 mL volumes by intraperitoneal injection. The total treatment cycle was 21 days and the treatment frequency was twice per week. The diet, mental state, and activities of tumor-bearing mice were observed daily. The tumor volume and bodyweight of mice were monitored every three days.

At 48 h after the last administration, the tumor-bearing mice were sacrificed, and the tumors were completely removed, anti-tumor efficacy was assessed by H&E and CD31 (abcam, Ab182981, 1:100) antigen staining and observation under a microscope (BA 400 Digital, Motic, LH, GER). The tumors were weighted with an electronic balance. The tumor inhibition rate was calculated as follows:

$$\text{tumor inhibition rate }(\%)=\frac{W_{\mathrm{control}}-W_{\mathrm{experimental}}}{W_{\mathrm{control}}}\times 100\%$$

where W_control_ is the average tumor weight of the control group, W_experimental_ is the average tumor weight of the experimental group.

### 2.10. Statistical analysis

Statistical data were analyzed using SPSS 17.0 and were summarized as the means ± standard deviation (SD). Comparisons between groups were performed using a one-way ANOVA test, and a *P* value less than .05 was considered statistically significant.

## 3. Results

### 3.1. Preparation, optimization, and characterization of PC-SLN

Based on the single-factor test, four main factors affecting the quality of PC-SLN were identified, namely the amount of CU + PTX (A), HSPC (B), DSPE-PEG2000 (C), and ultrasonic intensity (D). The L_9_(3^4^) orthogonal experiments were used to determine a stable and feasible technological formula, the results were shown in Table 1. And the result of range analysis was shown that factor A had the greatest impact on the comprehensive score, followed by factor D and factor C, factor B had the smallest impact, and the best prescription was A2B1C3D3. The analysis of variance showed that factor A factor C and factor D had a significant effect on the comprehensive score with *P* values of less than .05, while factor B had no significant effect (*P* values were more than .05), which was consistent with the results of the intuitive analysis. In brief, the optimal formulation was composed of CU 5 mg, PTX 2.5 mg, HSPC 70 mg, and DSPE-PEG2000 30 mg, ultrasonic intensity during preparation was 35%.

**Figure 1.**
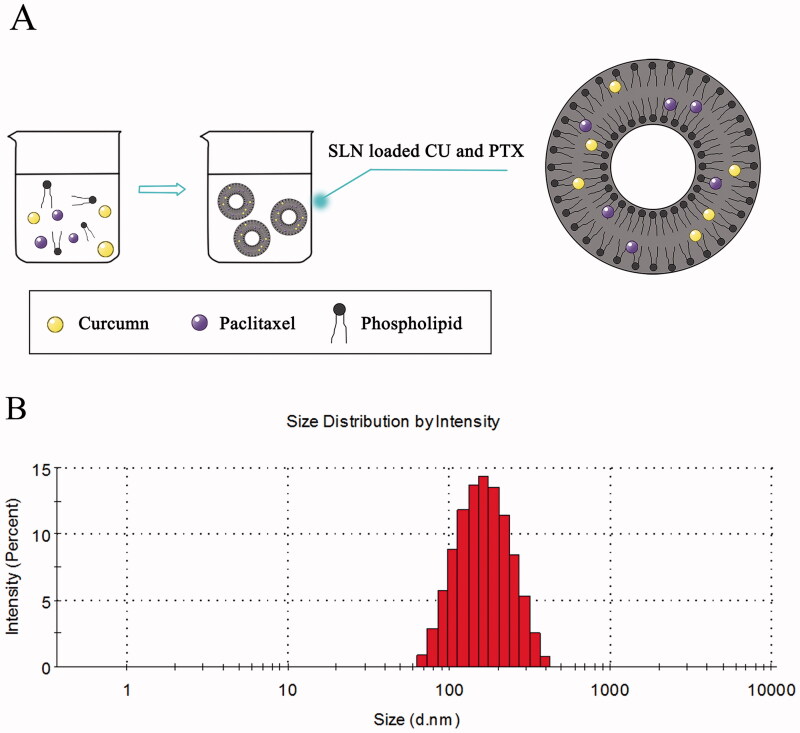
Design and size distribution of PC-SLN. (A) Schematic design of PC-SLN. (B) Size distribution spectrum as determined by dynamic light scattering.

Three batches of CU-SLN, PTX-SLN, and PC-SLN were prepared under optimized conditions. A schematic diagram of the PC-SLN structure and preparation process was illustrated in Figure 1(A). The related parameters, such as the zeta potential, EE%, size, and PDI, are shown in Table 2. The average PC-SLN size was 121.8 ± 1.69 nm with a 0.267 ± 0.023 PDI and zeta potential of −30.4 ± 1.25 mV. The EE% of CU and PTX in nanoparticles were 97.6 ± 1.63% and 93.5 ± 2.13%, respectively. Typical droplet size and distribution are shown in Figure 1(B).

**Table 2.** Characterization of the prepared SLN (*n* = 3).

Formulation	Mean diameter (nm)	PDI	zeta potential (mV)	EE (CU)%	EE (PTX)%
CU-SLN	88.40 ± 1.49	0.153 ± 0.015	−22.7 ± 1.05	98.3 ± 1.00	–
PTX-SLN	107.2 ± 2.93	0.254 ± 0.005	−27.0 ± 0.61	–	69.4 ± 2.68
PC-SLN	121.8 ± 1.69	0.267 ± 0.023	−30.4 ± 1.25	97.6 ± 1.63	95.8 ± 2.13

### 3.2. Cytotoxicity assay

**Figure 2.**
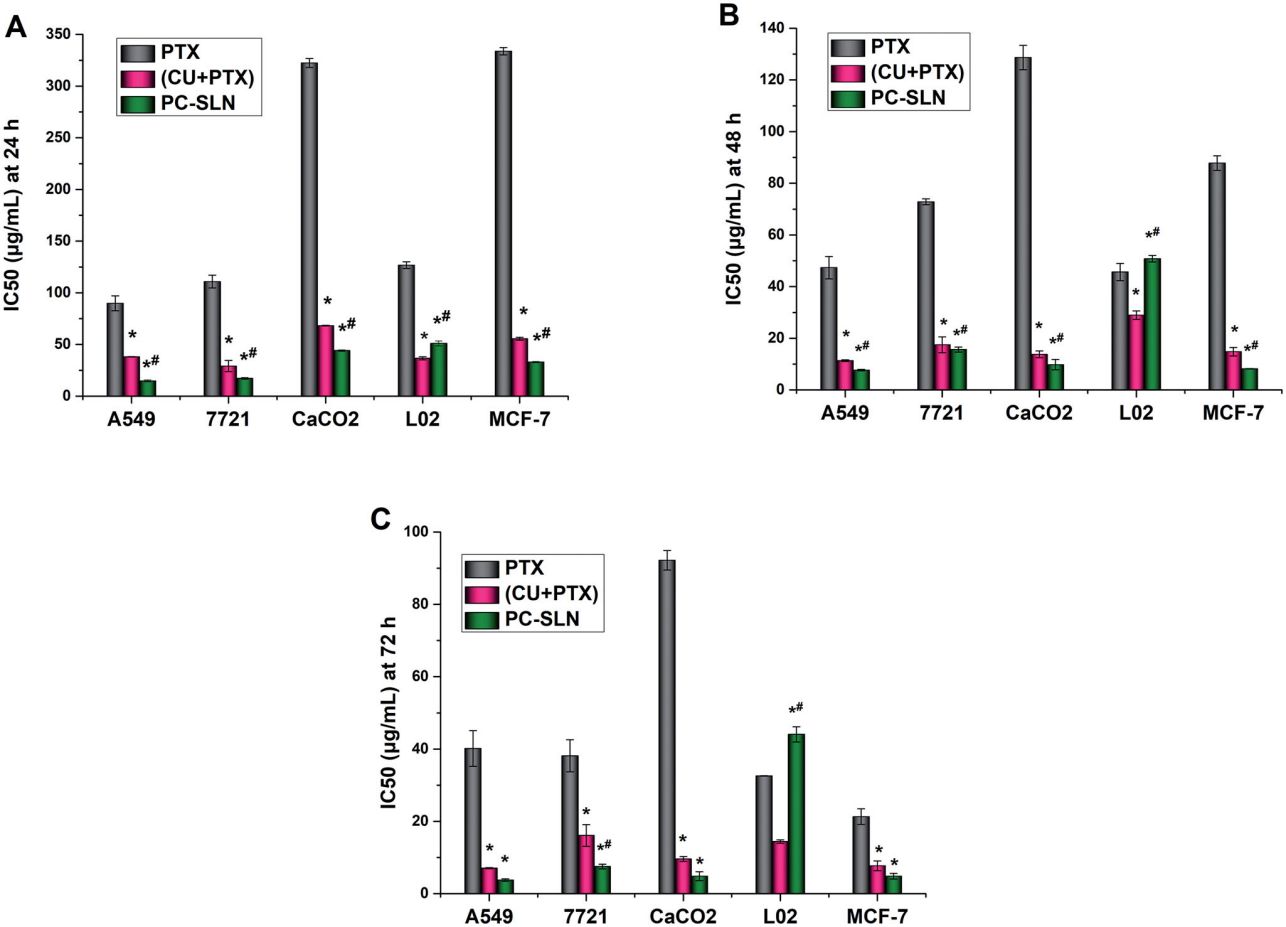
Cytotoxicity of PC-SLN. IC_50_ values of free PTX and the PTX of (CU + PTX) and PC-SLN on a panel of cell lines were determined at 24 h (A), 48 h (B) and 72 h (C) (means ± SD, *n* = 3). t test: *, *P* < .05, there was a significant difference compared with PTX; #, *P* < .05, there was a significant difference compared with (CU + PTX).

To further investigate the synergistic and attenuated effects of PC-SLNs, human liver cancer (SMMC-7721), lung cancer (A549), breast cancer (MCF-7), colon cancer (CaCO2), and normal liver (L02) cell lines were screened. The MTT method was used to investigate the inhibitory effects of PTX, (CU + PTX), and PC-SLN on proliferation in cell lines at 24 h, 48 h, and 72 h. For cancer cells, the smaller the IC_50_ value, the greater the inhibitory effect of the drug on cancer cell proliferation. In contrast, the larger the IC_50_ value in normal cells, the better the drug safety. The IC_50_ results (Figure 2) showed that the growth of the five cell lines was inhibited by the PTX, CU + PTX, and PC-SLN treatments. The toxicity of the three-drug groups on L02 cells (an immortalized normal liver cell line) increased as the time of action increased during 24–72 h. After 48 h and 72 h, the IC_50_ values of PC-SLN on L02 cells were increased by 1.11-fold and 1.35-fold compared with PTX, and by 1.75-fold and 3.05-fold compared with that of (CU + PTX), indicating that after CU and PTX were jointly prepared into PC-SLNs, the drug safety was improved. The inhibitory effect of the three drugs on four cancer cell lines increased as the time of action increased during 24–72 h. PC-SLN showed the highest inhibition intensity against each cancer cell at each time point, among which PC-SLN had the strongest inhibitory effect on A549 cells. After 24 h, 48 h, and 72 h, the IC_50_ values of PC-SLN on A549 cells were decreased by 6.08-fold, 6.16-fold, and 7.32-fold compared with PTX, and by 2.58-fold, 1.47-fold and 1.29-fold compared with that of (CU + PTX).

### 3.3. Cell apoptosis and cell cycle

**Figure 3.**
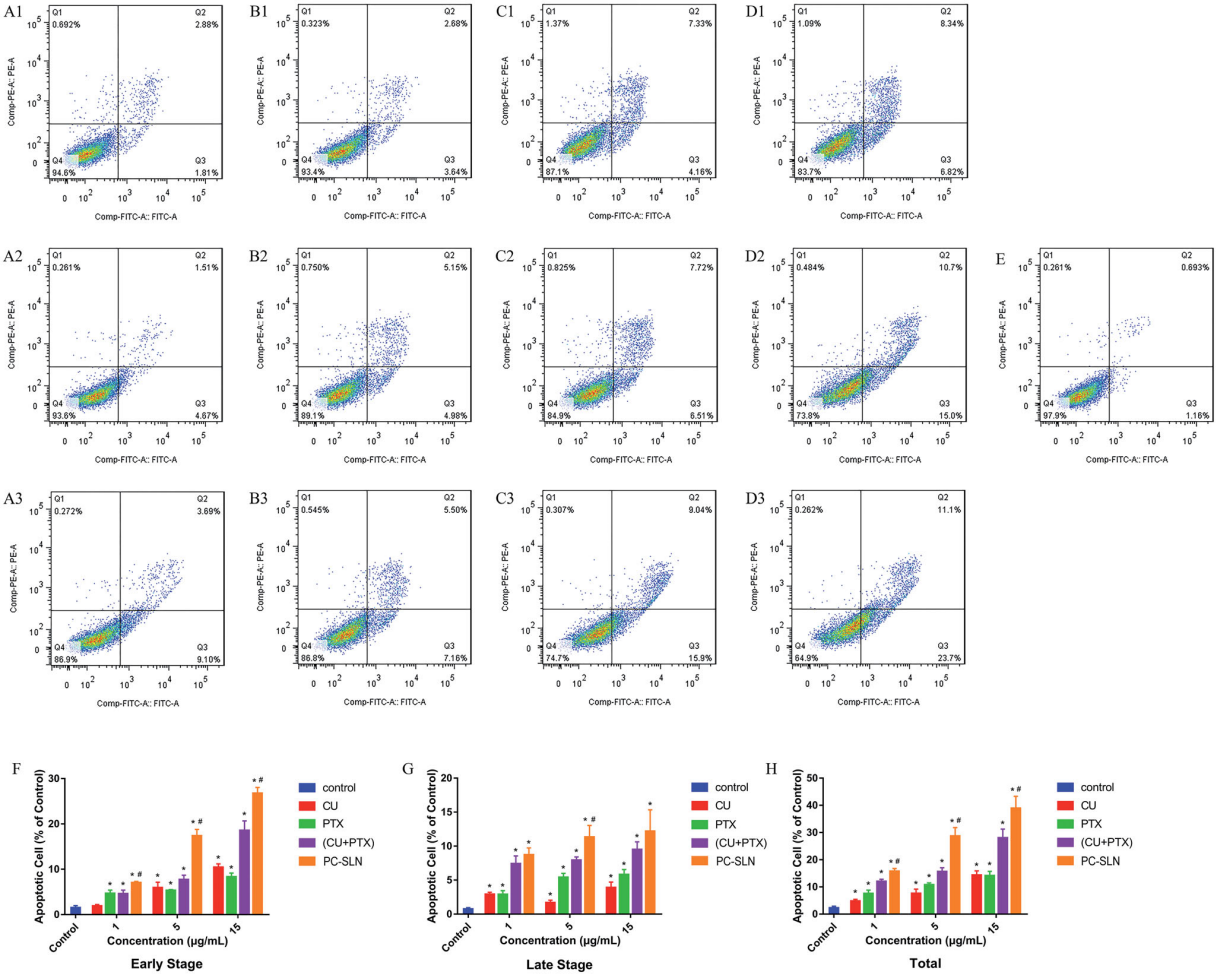
Induction of apoptosis in A549 cells by PC-SLN. Effects of CU (A), PTX (B), (CU + PTX) (C) and PC-SLN (D) at 1 μg/mL, 5 μg/mL and 15 μg/mL with the control on cell death were evidenced by annexin VFITC/PI double staining and FACS analysis. (F) Early apoptosis of CU, PTX, (CU + PTX) and PC-SLN in A549 cells at 48 h. (G) Late apoptosis of CU, PTX, (CU + PTX) and PC-SLN to A549 cells at 48 h. (H) Total apoptosis of CU, PTX, (CU + PTX) and PC-SLN to A549 cells at 48 h (means ± SD, *n* = 3). t test: *, *P* < .05, there was a significant difference compared with PTX; #, *P* < .05, there was a significant difference compared with (CU + PTX).

To discover whether the combination of CU and PTX can also induce apoptosis in A549 cells and whether PC-SLNs can enhance the apoptotic effect, A549 cells were treated with 1 μg/mL, 5 μg/mL, and 15 μg/mL of CU, PTX, (CU + PTX) and PC-SLN for 48 h. Flow cytometry revealed that the apoptotic effect was in a concentration-dependent manner. At doses of 1 μg/mL, 5 μg/mL, and 15 μg/mL, the apoptotic rates of CU, PTX, (CU + PTX) and PC-SLN on A549 cells were 4.75%, 7.58% and 14.30%; 7.51%, 10.72%, and 14.11%; 11.96%, 15.60%, and 28.02%; and 15.72%, 18.70% and 38.90%, respectively (Figure 3). (CU + PTX) could promoted the apoptotic effect of PTX on A549 cells, and PC-SLNs further enhanced the apoptosis of A549 cells induced by (CU + PTX).

**Figure 4.**
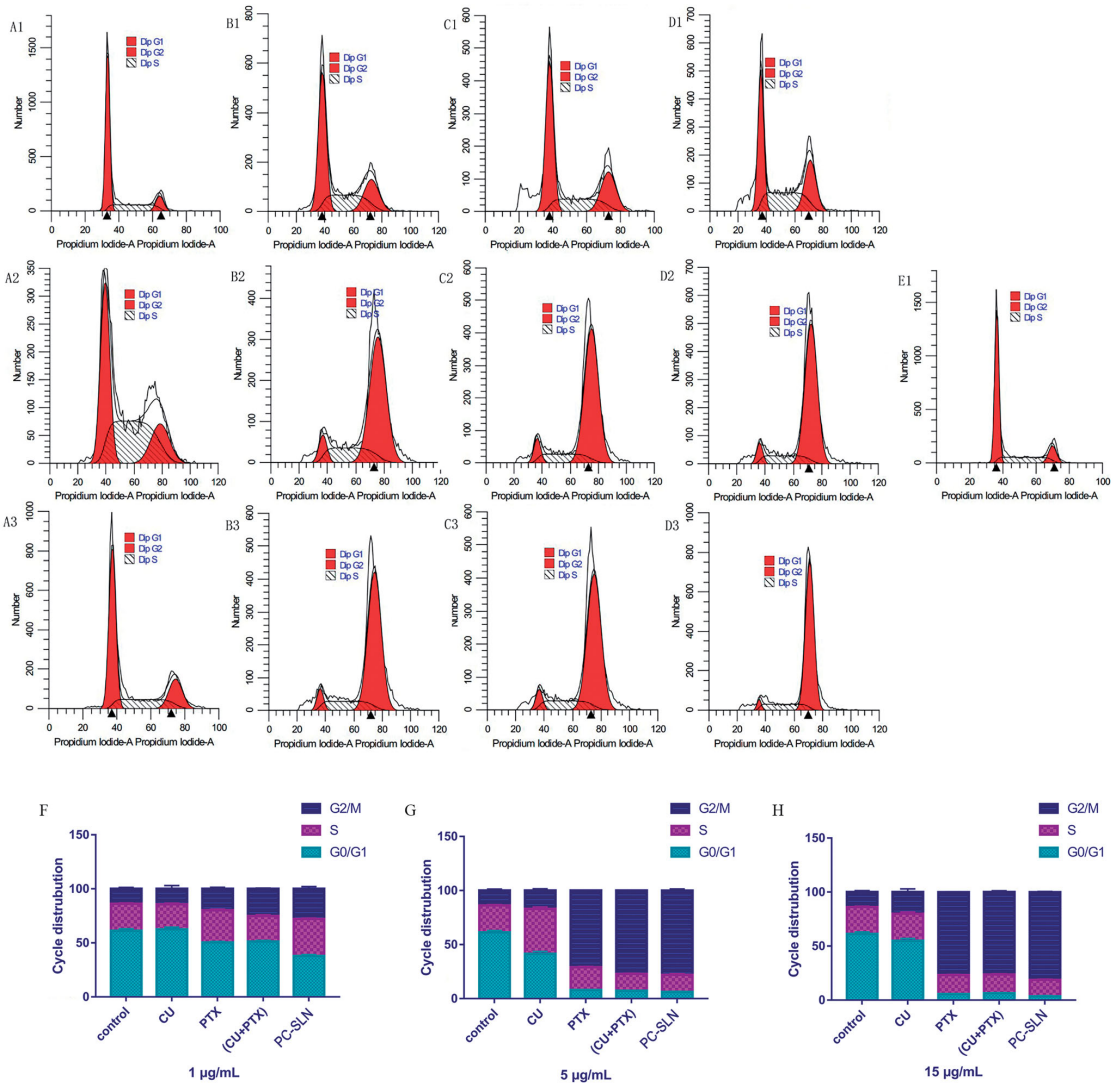
Effects of PC-SLN on cell cycle. Flow cytometry was performed to examine the effects of the cell cycle with CU (A), PTX (B), (CU + PTX) (C) and PC-SLN (D) at 1 μg/mL, 5 μg/mL and 15 μg/mL with the control (E) in A549 cells. Frequency distribution bar chart for G0/G1, S, and G2/M at doses of 1 μg/mL (F), 5 μg/mL (G), and 15 μg/mL (H). (means ± SD, *n* = 3).

The cell cycle results showed that compared to the control group, CU, PTX, (CU + PTX) and PC-SLN had a higher percentage of cells in the G2-M phase (*P* < .05). After 48 h of treatment, the number of cells in the G2-M phase in the 1 μg/mL PC-SLN group was 27.81%, which was 13.43%, 7.98%, and 2.65% more than that in the CU, PTX, and CU + PTX groups, respectively. The number of cells in the G2-M phase in the PC-SLN group with 5 μg/mL was 77.91%, which was 61.01%, 6.98%, and 0.88% more than that in the CU, PTX, and (CU + PTX) groups, respectively. The number of cells in the G2-M phase in the PC-SLN group with the 15 μg/mL was 81.13%, which was approximately 61.05%, 4.31%, and 4.83% more than that in the CU, PTX, and (CU + PTX) groups, respectively (Figure 4).

### 3.4. Protein expression

**Figure 5.**
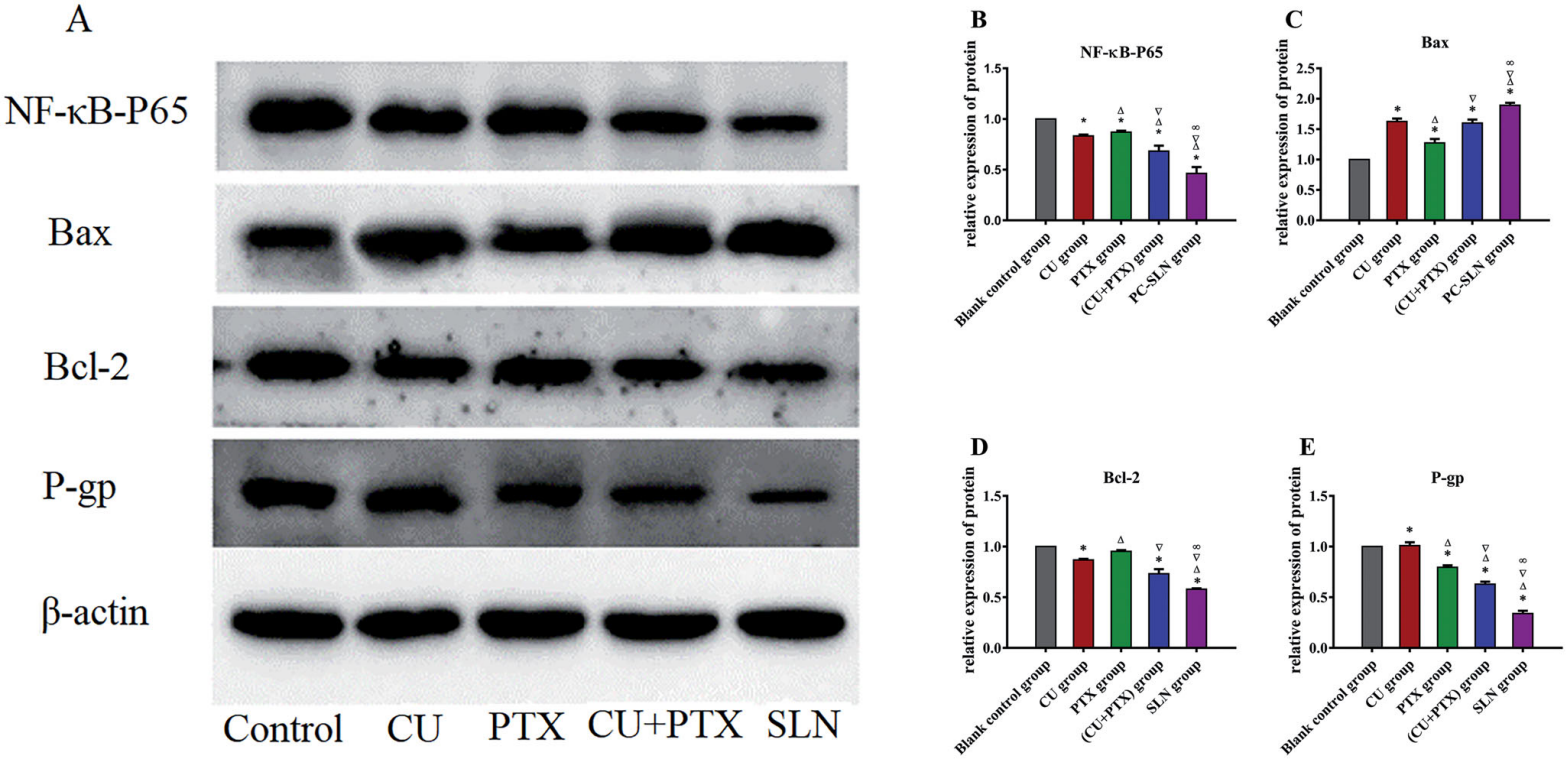
Effects of PC-SLN on biomarkers. (A) Effects of BA, BAD, and BAL on the expression of NF-κB-P65, Bax, Bcl-2 and P-gp by western blotting analysis at three concentrations in A549 cells for 48 h. Effects of CU, PTX, (CU + PTX) and PC-SLN on the value of NF-κB-P65 (B), Bax (C), Bcl-2 (D) and P-gp (E) in A549 cells for 48 h. t test: *, *P* < .05, compared with negative control group; △, *P* < .05, compared with the CU group; ▽, *P* < .05, compared with the PTX group; ∞, *P* < .05, compared with the CU + PTX group. (means ± SD, *n* = 3).

To determine whether PC-SLN or the combination treatment could promote apoptosis and block the cell cycle by inhibiting the expression of NF-κB, as CU and PTX did, western blotting was performed to detect the expression of NF-κB. The relative protein expression of NF-κB-P65, Bax, Bcl-2, and P-gp in the cells of each experimental group was detected and compared with that in the blank group. Following the western blotting results, 4 groups of drugs were applied to A549 cells for 48 h, and the corresponding protein expressions were further detected by western blotting (Figure 5(A)). Compared with the blank control group, the expression of Bax protein in the CU, PTX, CU + PTX, and PC-SLN groups increased. The expression of NF-κB-P65, Bcl-2, and P-gp proteins decreased. Compared with the four-drug groups, PC-SLN had the strongest promoting effect on the expression of Bax protein and more obviously inhibited NF-κB-P65, Bcl-2, and P-gp protein expressions; furthermore, PC-SLN had the strongest inhibitory effect on P-gp protein. The comparison results were significantly different (*P* < .05) (Figure 5(B)).

### 3.5. Pharmacokinetic studies

**Figure 6.**
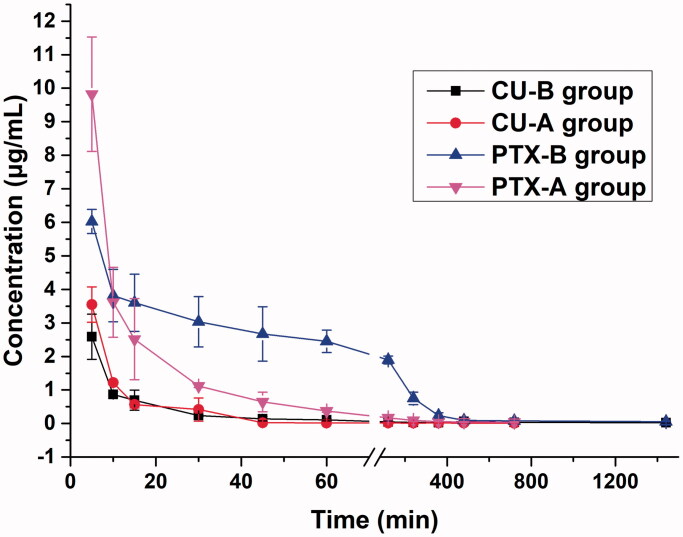
The plasma concentration-time curves of the combination (Group A) and SLNs (Group B) at the same doses. Data from different time points of each group are presented as the mean ± SD (*n* = 5).

To study the pharmacokinetic behaviors of PC-SLNs, rabbits were injected with PC-SLNs (16 mg/kg CU, 8 mg/kg PTX) (Group B) or a combination of free drugs at 8 mg/kg PTX and 16 mg/kg CU as the control (Group A). The plasma concentration-time curves of different groups are presented in Figure 6. The pharmacokinetic parameters were determined by non-compartmental analyses and are summarized in Table 3. The pharmacokinetic parameters were significantly changed with (CU + PTX) and PC-SLN. PC-SLN exhibited a higher AUC_0-T_ (CU: 1.40-fold; PTX: 2.88-fold), MRT_0-T_ (CU: 6.94-fold; PTX: 2.51-fold), and t_1/2z_ (CU: 5.62-fold; PTX: 6.46-fold) than that of (CU + PTX). PC-SLNs also had lower CLz than that of (CU + PTX) (CU: 2.82-fold; PTX: 2.87-fold).

**Table 3.** Pharmacokinetic parameters of PC-SLN and combination powder in rabbits (*n* = 5).

Pharmacokinetic parameters	Unit	Combination at 16 mg/kg of CU and 8 mg/kg of PTX (Group A)		PC-SLN (16 mg/kg of CU, 8 mg/kg of PTX) (Group B)	
		CU	PTX	CU	PTX
AUC_0-T_	mg/L*min	65.48	224.00	91.49	644.78
MRT_0-T_	min	50.73	78.62	351.99	197.86
t_1/2z_	min	635.31	207.073	3572.97	1337.00
CLz	L/min/kg	0.11	0.066	0.039	0.023
Cmax	mg/L	3.553	9.821	2.599	6.245

### 3.6. Treatment effect in tumor-bearing mice

**Figure 7.**
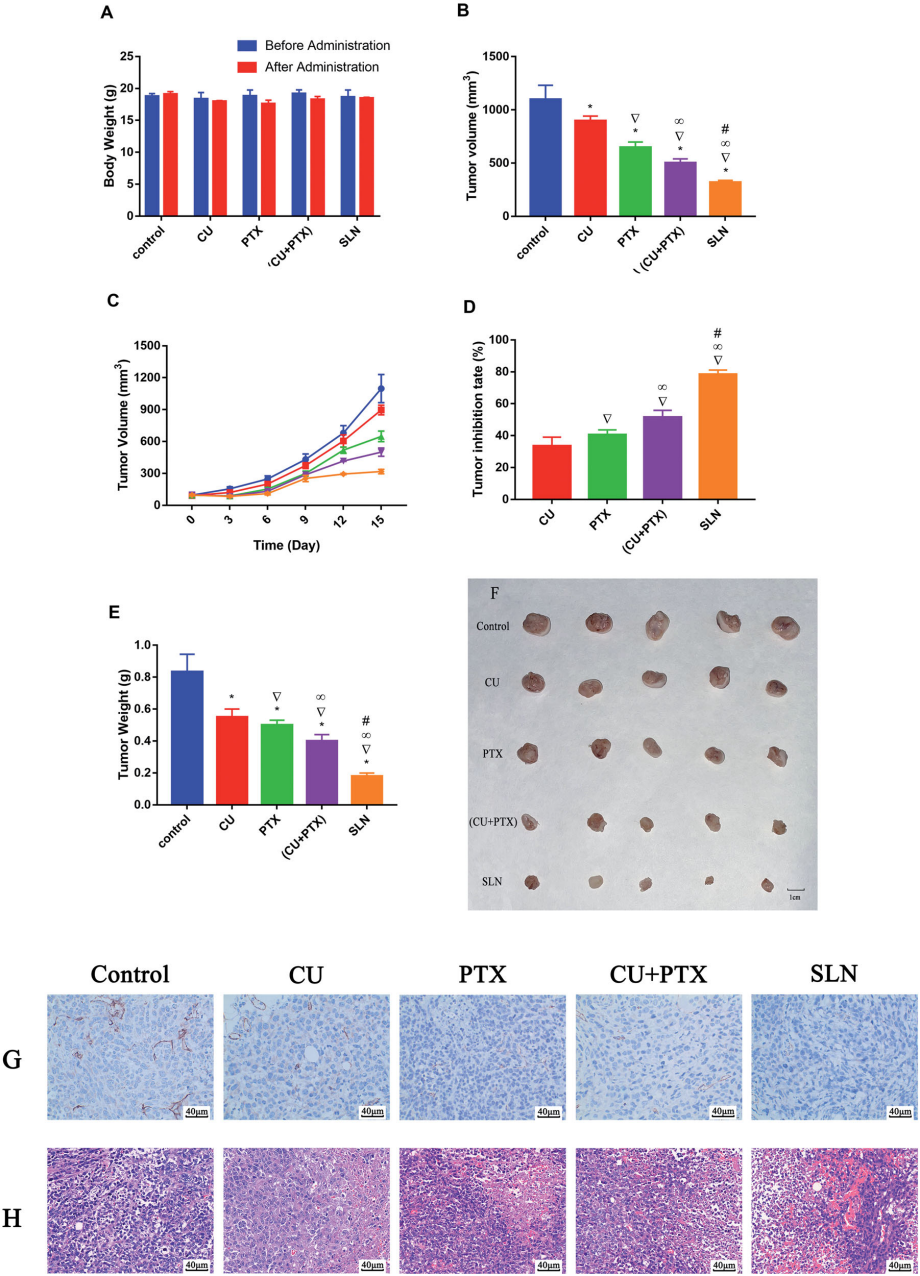
Antitumor efficacy and toxicity of PC-SLN *in vivo* in nude mice bearing A549 tumor xenografts. (A) Changes in body weight (g) before and after treatment with CU, PTX, (CU + PTX) and PC-SLN in nude mice. (B) Tumor volume of A549-bearing nude mice treated with CU, PTX, (CU + PTX) and PC-SLN on Day 15. (C) Changes in tumor volume in different groups of nude mice. (D) Tumor inhibitory rates (%) of A549-bearing nude mice treated with CU, PTX, (CU + PTX) and PC-SLN on Day 15. (E) Tumor weights (g) of A549-bearing nude mice treated with CU, PTX, (CU + PTX) or PC-SLN on Day 15, after which the mice were humanely sacrificed. (F) Photograph of tumors on Day 15 when the mice were humanely sacrificed. (G) The results of CD31 in tumor tissues were analyzed by immunohistochemistry. (H) The results of tumor mass H&E staining (means ± SD, *n* = 5). *t*-test: *, *P* < .05, compared with the negative control group; ▽, *P* < .05, compared with the CU group; ∞, *P* < .05, compared with the PTX group; #, *P* < .05, compared with the CU + PTX group.

An animal model was established with nude mice bearing A549 cells to further investigate the antitumor activity of PC-SLNs *in vivo*. No mice died during the experiments, and there was no significant difference in the weight of each group compared with that of the control group at the end of the experiment. (Figure 7(A)). All treatment methods prevented the increase in tumor volume to different degrees (Figure 7(B)). At the end of the trial, the mean tumor volume in the PC-SLN group was 28.91% of that in the control group, which was significantly smaller than that in either the CU group (*P* < .05), PTX group (*P* < .05), or, (CU + PTX) group (*P* < .05). The tumor inhibition rate of PC-SLN was 78.42%, while that of CU, PTX, or, (CU + PTX) was 33.57% (*P* < .05), 40.53% (*P* < .05), and 51.56% (*P* < .05), respectively (Figure 7(D)). The immunohistochemical reaction of CD31 in the PC-SLN group was the weakest, about 15% (Figure 7(G)). HE staining showed that nearly 75% of cancer cell necrosis was detected in the PC-SLN-treated group, compared to 15% in CU, 30% in PTX, and 65% in (CU + PTX) (Figure 7(H)).

## 4. Discussion

The therapeutic potential of CU and PTX could be improved by encapsulating the drugs with SLNs. Previous studies, which evaluated the synergetic effects of combined CU and PTX treatments and the best proportion of CU and PTX to achieve these effects, had a beneficial synergistic effect on cancer cells and an excellent safety profile in found that CU and PTX with a mass ratio of 2:1 had a beneficial synergistic effect on cancer cells and an excellent safety profile in L02 (Li, 2019; Feng et al., 2020). In this study, both PTX and CU were encapsulated in SLNs at a mass ratio of 2:1, which were composed of HSPC, DSPE-PEG2000, and PVPk15.

The results of single-factor experiments showed that with the addition of PVPk15, the EE% and DL% of CU and PTX in PC-SLN increased, and the particle size became more uniform. This may be due to the association between drug molecules and PVPk15, which can inhibit the crystallization of CU or PTX during the preparation process, thus obtaining PC-SLNs with a small uniform particle size. As the membrane material of SLN, HSPC is helpful to improve the stability of SLN, while DSPE-PEG2000 can realize the long-term circulation of the drug (Behbahani et al., 2017; Zhang et al., 2019). With the increase of the amount of DSPE-PEG2000 or HSPC, the EE% of CU and PTX increased, while with the increase of the amount of (CU + PTX), the EE% and DL% of CU and PTX increased first and then decreased, which may be caused by too much membrane material redundant empty SLNs. When the probe-type ultrasound is used to prepare SLN, the energy emitted by the probe may be unevenly distributed in the container, resulting in homogenization of the particles to different degrees (Behbahani et al., 2017). With the increase of ultrasonic intensity, the particle size of PC-SLN decreased, and the PDI first decreased and then increased. Excessive sonication may damage the structure of the SLN, generate SLN fragments, cause an increase in PDI. Based on the results of the single-factor investigation, the factors that have a greater impact on the preparation of (CU + PTX)-SLN were selected, including the amount of (CU + PTX), the amount of HSPC, the amount of DSPE-PEG2000, and the ultrasonic intensity. The optimal formulation for preparing PC-SLN was screened out by orthogonal experimental design, and the formulation was repeated in three batches to verify that the formulation process was stable and reliable.

The EE% is an important index for evaluating a successful coloaded drug delivery system (Xie et al., 2017). The favorable EE% of PC-SLN (CU and PTX were both more than 95%) indicates that the two drugs are encapsulated in a predetermined ratio (Figure 1(A)). Interestingly, the EE% of PTX was significantly increased after CU was added to the coloaded PC-SLNs compared with the encapsulated PTX-SLNs alone. This demonstrates that in pharmaceutical applications, CU may be a good material to facilitate encapsulations, namely, CU is a good co-loading partner companion for PTX. In addition, the negative charge on the surface of PC-SLN (-30.4 ± 1.25 mV) helps to achieve good homogeneity in the liquid environment and the stability of PC-SLN. Moreover, uniform and appropriate particle size (121.8 ± 1.69 nm) and small PDI (0.267 ± 0.023) contribute to the improvement of drug efficacy and pharmacokinetic behavior (Hong and Nam, 2014; Maritim et al., 2021).

*In vitro* research is very important for determining the sensitivity of cancer cells to the drugs being studied. Compared with PTX alone, significantly lower IC_50_ of the PTX was achieved after combination, resulting in very efficient treatments of the A549, 7721, CaCO2, and MCF-7 cancer lines at a low dose. Among them, CU may offer an effective chemosensitizer to improve the therapeutic index of PTX, which is consistent with previously reported studies (Calaf et al., 2018; Ashrafizadeh et al., 2020; Lee et al., 2020). After CU and PTX were jointly prepared into PC-SLNs, the inhibitory effect of PTX on cancer cells was further improved, among which the inhibitory effect on A549 lung cancer cells was strongest, suggesting that PC-SLNs have a high clinical therapeutic value for lung cancer treatments (Figure 2). In addition, PC-SLNs also reduced the cytotoxicity that was caused by the combination of CU + PTX.

Apoptosis plays an important role in the proliferation, drug resistance, and metastasis of tumors. PTX and CU have been shown to promote cancer cell apoptosis through multiple pathways (Miller et al., 2013; Endo et al., 2020). Compared with PTX alone, (CU + PTX) resulted in a higher rate of apoptosis. Besides, compared to (CU + PTX), PC-SLNs can promote the apoptosis of tumor cells more effectively and in a concentration-dependent manner, which may be related to the difference in the permeability of PTX and CU. Recent studies have shown that the permeability of drugs influences cell apoptosis, while CU has a greater permeability than that of PTX (Hope et al., 2019; Abbasi et al., 2021). Therefore, CU might be released from PC-SLNs to regulate the relevant signaling pathways and cause cancer cells to be sensitive to PTX. As a result of the cell cycle, the mechanism by which PC-SLN promotes A549 apoptosis may be to inhibit the unlimited proliferation of cancer cells by blocking the cell cycle in the G2-M phase.

The PI3K/Akt pathway is an intracellular signal transduction pathway with enzymatic activity that regulates the proliferation and survival of cancer cells (Fumarola et al., 2014). Akt, as the core of this pathway, regulates the expression of target genes by phosphorylating downstream substrates (Mayer and Arteaga, 2016; Ippen et al., 2019). Studies have shown that CU or PTX can promote cancer cell apoptosis by inhibiting the phosphorylation of Akt in the PI3K/Akt pathway and then upregulating Bax and downregulating the expression of Bcl-2 protein (Hwang et al., 2016). The western blot analysis results showed that PC-SLNs had the strongest effect on promoting the expression of Bax protein and inhibiting the expression of Bcl-2, which was consistent with the apoptosis results. Overexpressing P-gp can mediate the efflux of drugs, thus leading to a significant reduction in the intracellular drug concentrations (Zhang et al., 2016; Ding et al., 2017). We found that PC-SLNs effectively downregulated the expression of the P-gp protein (Figure 5), suggesting that PC-SLNs can reverse MDR via the P-gp pathway. To elucidate how PC-SLN regulates the P-gp pathway, the NF-κB signal transduction pathway attracted our attention. NF-κB is active in most tumor cells, and regulatory genes are related to cell transformation, proliferation, survival, invasion, angiogenesis, metastasis and chemical resistance (Zhang et al., 2016; Zakaria et al., 2018). It has been reported that activated NF-κB can bind to the κB binding sites located upstream of the mdr1 transcription start site, resulting in increased expression of the mdr1 gene (Monisha et al., 2016).Thus, we believe that PC-SLN may inhibit the activity of NF-κB in the same way as P-gp.

Well-defined pharmacokinetic characteristics are helpful to achieve the expected therapeutic effect. PC-SLN prolonged the residence time, reduced the clearance, and improved the bioavailability of both CU and PTX. In PC-SLNs, DSPE-PEG2000 can coat the SLN surface through hydrophobic interactions, while PEG macromolecules extend on the lipid membrane surface and are highly hydrated in the water environment; this forms a thick hydration membrane protective layer on the SLN surface to reduce the mutual aggregation of SLNs and various components in blood, achieving the steric stability of SLNs (Wang et al., 2019). Furthermore, the negative zeta-potential on PC-SLNs is conducive to the stability of nanoparticles, which can prevent them from combining with plasma proteins and prolonging their circulation (Zhang et al., 2019).

To the best of our knowledge, although many new formulations loaded with both PTX and CU have emerged, including liposomes, PLA-TPGS and magnetic PLGA NPs, most of the pharmacodynamic results in these studies are based on *in vitro* evaluation only (Cui et al., 2016; Liu et al., 2016; Alemi et al., 2018). In the present study, we found that the synergistic effect of CU and PTX *in vitro* could also be observed *in vivo* and after CU and PTX were jointly prepared into PC-SLNs, this synergistic effect was enhanced. The results of *in vivo* anti-tumor trials have shown that the growth of tumors was controlled by PC-SLN and efficacy increased with time. The results of H&E staining and immunohistochemistry once again demonstrated that PC-SLN inhibits lung cancer cell and tumor proliferation, which was consistent with the results of the cytotoxicity, apoptosis, and western blotting on A549 *in vitro*. PC-SLNs are potential antineoplastic drugs that are effective in treating lung cancer. The overall efficacy of the PC-SLN codelivery system on lung cancer was achieved through improved pharmacokinetic behavior and combined regulation of the NF-κB pathway by CU and PTX.

## 5. Conclusion

Based on the data in this study, common problems, such as low EE% and poor stability of co-loaded drugs, were solved by PC-SLNs, while as a co-loading partner, CU can improve pharmaceutical profile of PC-SLNs. At the same time, PC-SLNs can increase the efficacy of coadministered drugs and reduce drug toxicity. Furthermore, the *in vivo* experiment shows that PC-SLNs have proper pharmacokinetic behavior, and the synergistic effect of PTX and CU *in vitro* can reappear *in vivo* via PC-SLNs. This study provides a new direction for the development of combined anticancer therapy and finally leads to more effective and less toxic anticancer therapies.

## Data Availability

The datasets used or analyzed during the current study are available from the corresponding author on reasonable request.
